# Implications of deduplication on the detection rates of multidrug-resistant organism (MDRO) in various specimens: insights from the hospital infection surveillance program

**DOI:** 10.1186/s13756-024-01408-2

**Published:** 2024-05-21

**Authors:** Zhanjie Li, Dan Zhu, Xiaoju Ma, Feng Zang, Weihong Zhang, Can Luo, Chuanlong Zhu, Wensen Chen, Ping Zhu

**Affiliations:** 1 Department of Infection Control, The First Affiliated Hospital of Nanjing Medical University, Nanjing, Jiangsu 210029 China; 2 Department of Hospital Infection Management, Huabei Petroleum Administration Bureau General Hospital, Cangzhou, Hebei 062550 China; 3 Department of Hospital Acquired Infection Control and Public Health Management, The Seventh Affiliated Hospital, Sun Yat-Sen University, Shenzhen, Guangdong 518107 China; 4 Department of Infectious Disease, The First Affiliated Hospital of Nanjing Medical University, Nanjing, Jiangsu 210029 China; 5 Department of Epidemiology and Biostatistics, School of Public Health, Xi’an Jiao Tong University Health Science Center, Xi’an, Shaanxi 710061 China; 6 Department of Quality Management, The First Affiliated Hospital of Nanjing Medical University, Nanjing, Jiangsu 210029 China

**Keywords:** Deduplicate, MDRO, Detection rates, Different specimens, Implications

## Abstract

**Background:**

Currently, different guidelines recommend using different methods to determine whether deduplication is necessary when determining the detection rates of multidrug-resistant organisms (MDROs*)*. However, few studies have investigated the effect of deduplication on MDRO monitoring data. In this study, we aimed to investigate the influence of deduplication on the detection rates of MDROs in different specimens to assess its impact on infection surveillance outcomes.

**Methods:**

Samples were collected from hospitalized patients admitted between January 2022 and December 2022; four types of specimens were collected from key monitored MDROs, including sputum samples, urine samples, blood samples, and bronchoalveolar lavage fluid (BALF) samples. In this study, we compared and analysed the detection rates of carbapenem-resistant *Klebsiella pneumoniae* (CRKP), carbapenem-resistant *Escherichia coli* (CRECO)*,* carbapenem-resistant *Acinetobacter baumannii* (CRAB)*,* carbapenem-resistant *Pseudomonas aeruginosa* (CRPA)*,* and methicillin-resistant *Staphylococcus aureus* (MRSA) under two conditions: with and without deduplication.

**Results:**

When all specimens were included, the detection rates of CRKP, CRAB, CRPA, and MRSA without deduplication (33.52%, 77.24%, 44.56%, and 56.58%, respectively) were significantly greater than those with deduplication (24.78%, 66.25%, 36.24%, and 50.83%, respectively) (all *P* < 0.05). The detection rates in sputum samples were significantly different between samples without duplication (28.39%, 76.19%, 46.95%, and 70.43%) and those with deduplication (19.99%, 63.00%, 38.05%, and 64.50%) (all *P* < 0.05). When deduplication was not performed, the rate of detection of CRKP in urine samples reached 30.05%, surpassing the rate observed with deduplication (21.56%) (*P* < 0.05). In BALF specimens, the detection rates of CRKP and CRPA without deduplication (39.78% and 53.23%, respectively) were greater than those with deduplication (31.62% and 42.20%, respectively) (*P* < 0.05). In blood samples, deduplication did not have a significant impact on the detection rates of MDROs.

**Conclusion:**

Deduplication had a significant effect on the detection rates of MDROs in sputum, urine, and BALF samples. Based on these data, we call for the Infection Prevention and Control Organization to align its analysis rules with those of the Bacterial Resistance Surveillance Organization when monitoring MDRO detection rates.

## Background

In the past century, antibacterial agents have played key roles in the fight against various infectious diseases, but the increasing prominence of multidrug-resistant organisms (MDROs) has brought substantial challenges in terms of clinical infection treatment [1–4]. Patients who develop MDRO infections are more difficult to treat, suffer more pain, recover more slowly, and have longer hospital stays, higher hospital costs, and even increased clinical mortality compared to patients infected with non-MDROs [5–7]. Strategies to slow the development of drug resistance in bacteria and prevent the transmission of drug-resistant organisms have attracted widespread interest internationally, as drug resistance has become an important obstacle for most clinical medical staff and management departments at all levels; this obstacle will have to be addressed to improve the quality of medical care [8, 9].

In 2022, 13 departments in China collaborated to form the Notice of the National Action Plan for Combating Antimicrobial Resistance (2022–2025) [10]. The plan outlines the general strategies for combating antimicrobial resistance: adhering to the principle of putting prevention first, combining prevention with treatment and comprehensive policies, focusing on the outstanding problems of antimicrobial drug resistance, and improving the prevention and control of infection in medical institutions and the clinical monitoring systems used for antimicrobial drugs. Unified and standard monitoring of bacterial drug resistance is important. In 2015, the Health Commission of the People's Republic of China released 13 quality control indicators for hospital infection management [11]. Among these indicators, the MDRO detection rate is an important index.

However, the current monitoring requirements for MDRO detection rates are not consistent in the fields of infection control and microbiology. For example, quality control indicators require that all isolated strains be included in MDRO detection rate statistics (i.e., without deduplication), whereas the China Antimicrobial Surveillance Network (CHINET) [12] requires that duplicate strains be excluded (i.e., deduplication).

When different standards are utilized, MDRO monitoring data vary greatly. Previous studies have demonstrated the impact of whether "intermediary" is considered "drug resistance" in MDRO monitoring statistics [13]. There have also been studies investigating the influence of different deduplication methods on MDRO monitoring data [14]. However, few studies have investigated the effect of deduplication on MDRO monitoring data.

Based on the above contradiction, in this study, we aimed to compare and analyse the similarities and differences in MDRO detection rates in different samples with or without deduplication to clarify the impact of deduplication on MDRO monitoring data and provide a reference for further unifying relevant monitoring standards.

## Methods

### Study samples

**Fig. 1:**
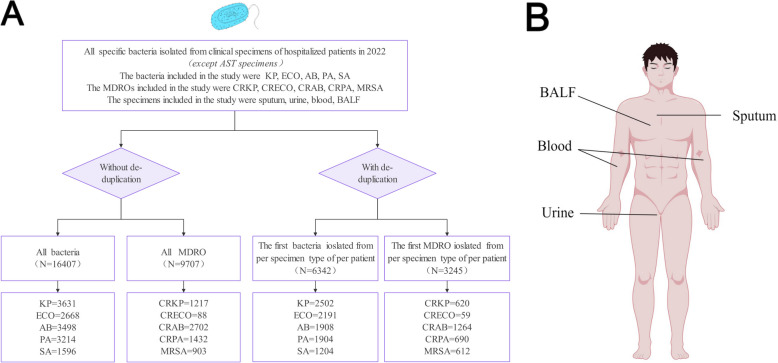
Overall screening process adopted in this research. **A** The flowchart shows the sample exclusion and inclusion criteria. **B** The anatomical distributions of the specimen types included in this study are shown. Note: AST, active surveillance test; KP, *Klebsiella pneumoniae;* ECO, *Escherichia coli;* AB, *Acinetobacter baumannii;* PA, *Pseudomonas aeruginosa; S*A, *Staphylococcus aureus*; MDRO, multidrug-resistant organism; CRKP, carbapenem-resistant *Klebsiella pneumoniae*; CRECO, carbapenem-resistant *Escherichia coli*; CRAB, carbapenem-resistant *Acinetobacter baumannii*; CRPA, carbapenem-resistant *Pseudomonas aeruginosa*; MRSA, methicillin-resistant *Staphylococcus aureus*; BALF, bronchoalveolar lavage fluid

This study focused on samples collected from inpatients at the First Affiliated Hospital of Nanjing Medical University (a Grade-A tertiary hospital with 4500 beds) from January 2022 to December 2022. The MDROs were isolated from the clinical microbiology laboratory, excluding specimens obtained from active surveillance testing (AST). The dataset included a total of 16,407 strains of bacteria, with 9707 strains classified as MDROs. Specifically, there were 3631 strains of *Klebsiella pneumoniae* (KP)*,* 1217 strains of carbapenem-resistant *Klebsiella pneumoniae* (CRKP)*,* 2668 strains of *Escherichia coli* (ECO)*,* 88 strains of carbapenem-resistant *Escherichia coli* (CRECO), 3498 strains of *Acinetobacter baumannii* (AB), 2702 strains of carbapenem-resistant *Acinetobacter baumannii* (CRAB), 3214 strains of *Pseudomonas aeruginosa* (PA), 1432 strains of carbapenem-resistant *Pseudomonas aeruginosa* (CRPA), 1596 strains of *Staphylococcus aureus* (SA) and 903 strains of methicillin-resistant *Staphylococcus aureus* (MRSA). Please refer to Fig. 1 for details. The Ethics Committee of the First Affiliated Hospital of Nanjing Medical University approved this study (2019-SR-075).

### Research methods

The Xinglin Real-Time Nosocomial Infection System was utilized to collect data from the samples. The samples from the key monitored MDROs were categorized into four types: sputum samples, urine samples, blood samples, and bronchoalveolar lavage fluid (BALF) samples. Analysis and comparison of the detection rates of CRKP, CRECO, CRAB, CRPA, and MRSA were conducted, both with and without deduplication.

### Deduplication method

Deduplication is defined as the selection and utilization of the first bacterium and MDRO isolated from each specimen type in each patient. That is, if the same MDRO was detected multiple times in the same sample from a single patient, the MDRO was considered only one strain. After deduplication, the dataset included 6342 bacterial strains and 3245 MDRO strains, including 2502 KP strains, 620 CRKP strains, 2191 ECO strains, 59 CRECO strains, 1908 AB strains, 1264 CRAB strains, 1904 PA strains, 690 CRPA strains, 1204 SA strains, and 612 MRSA strains. Figure 1 shows the details.

### Formula for determining MDRO detection rates

The detection rate of MDROs was calculated as follows: MDRO detection rate = (number of cases of specific multidrug-resistant bacteria in hospitalized patients/number of cases of specific bacteria in hospitalized patients in the same period) × 100%.

### Bacterial identification and drug sensitivity test

An automated VITEK 2® Compact system (bioMérieux, Marcy l’Etoile, France) was utilized for bacterial identification. Antimicrobial susceptibility testing was performed by VITEK® 2 (bioMérieux, Marcy l’Etoile, France) or the paper disk diffusion method (Oxide, United Kingdom) according to the manufacturer’s instructions. Zone diameter and minimum inhibitory concentration breakpoints were interpreted according to the 2021 edition of the Clinical and Laboratory Standards Institute (CLSI) [15]. CRKP and CRECO were defined as organisms satisfying any of the following conditions: (1) resistance to any carbapenem antimicrobial agent, such as imipenem, meropenem, ertapenem, or doripenem. CRPA and CRAB are defined as isolates that are resistant to any carbapenem antimicrobial agent, such as imipenem, meropenem, or doripenem [16]. MRSA is defined as *S. aureus* that is resistant to oxacillin or cefoxitin [17]. Quality control strains from the Clinical Testing Center of the National Health and Family Planning Commission, including *E. coli* (ATCC 25922), *K. pneumoniae* (ATCC 700603), *S. aureus* (ATCC 25923), *A. baumannii* (ATCC 19606), and *P. aeruginosa* (ATCC 278553), were used.

### Statistical analysis

The data are presented as frequencies (percentages). Descriptive statistics were obtained using either the chi-square test or Fisher's exact test, as appropriate. Analyses were performed with Statistical Product and Service Solutions (SPSS), version 23.0 (IBM Corp. Armonk, NY, USA), and images were generated by EdrawMax and Figdraw (https://www.figdraw.com/static/index.html, ID:ASUIR6773f). A *P* value < 0.05 indicated a statistically significant difference.

## Results

### Differences in deduplication requirements among relevant standards, guidelines, and monitoring systems

The quality control indicators and the Guidelines for Implementation in Infection Control require no deduplication, while the standards NHSN and JANIS require deduplication. CHINET, GLASS, CAESAR-Net, and EARS-Net in microbiology all require deduplication. For more details, please refer to Table 1.

**Table 1:** Differences in deduplication among relevant standards, guidelines, and monitoring systems

	Quality control indicators [11]	Guidelines for the implementation [18]	Standard [19]	CHINET [12]	GLASS [20]	CAESAR-Net [21]	EARS-Net [21]	NHSN [22]	JANIS [14]
Issuing country/institution	CHINA	CHINA	CHINA	CHINA	WHO	WHO	ECDC	AMERICA	JAPAN
Release time	2015	2021	2023	Every year	Every year	Every year	Every year	Every year	Every year
Deduplication	No	No	Yes	Yes	Yes	Yes	Yes	Yes	Yes

Quality control indicators, 13 quality control indicators of infection control; Guidelines for Implementation, Guidelines for the Implementation of Basic Data Set and Quality Control Index Set of Nosocomial Infection Monitoring (2021 Edition); Standard, Standard for Healthcare-associated Infection Surveillance; CHINET, China Antimicrobial Surveillance Network, *GLASS* Global Antimicrobial Resistance Surveillance System, *CAESAR-Ne* Central Asian and Eastern European Surveillance of Antimicrobial Resistance network, EARS-Net, The European Antimicrobial Resistance Surveillance Network, *NHSN* National Healthcare Safety Network, *JANIS* Japan Nosocomial Infections Surveillance, *WHO* World Health Organization, *ECDC* European Centers for Disease Control and Prevention

### Comparative analysis of MDRO detection rates with and without deduplication of all samples

The detection rates of CRKP, CRAB, CRPA, and MRSA in all specimens were notably greater when deduplication was not performed (33.52%, 77.24%, 44.56%, and 56.58%, respectively) than when it was conducted (24.78%, 66.25%, 36.24%, and 50.83%, respectively). These differences were statistically significant (all *P* < 0.05). The detection rate of CRECO without deduplication (3.30%) was slightly greater than that with deduplication (2.70%), but the difference was not statistically significant (*P* > 0.05). For more details, please refer to Table 2.

**Table 2:** Comparative analysis of MDRO detection rates with and without deduplication in all samples

MDRO		Without deduplication	With deduplication	X^2^	P
CRKP	CRKP	1217	620	53.890	< 0.001
	KP	3631	2502		
	Detection rate	33.52%	24.78%		
CRECO	CRECO	88	59	1.504	0.220
	ECO	2668	2191		
	Detection rate	3.30%	2.70%		
CRAB	CRAB	2702	1264	76.399	< 0.001
	AB	3498	1908		
	Detection rate	77.24%	66.25%		
CRPA	CRPA	1432	690	34.065	< 0.001
	PA	3214	1904		
	Detection rate	44.56%	36.24%		
MRSA	MRSA	903	612	9.133	0.003
	SA	1596	1204		
	Detection rate	56.58%	50.83%		

*CRKP* Carbapenem-resistant Klebsiella pneumoniae, *KP* Klebsiella pneumoniae, *CRECO* carbapenem-resistant Escherichia coli, *ECO* Escherichia coli, *CRAB* carbapenem-resistant Acinetobacter baumannii, *AB* Acinetobacter baumannii, *CRPA* Carbapenem-resistant Pseudomonas aeruginosa, *PA* Pseudomonas aeruginosa, *MRSA* methicillin-resistant Staphylococcus aureus, *SA* Staphylococcus aureus

### Comparative analysis of the detection rates of MDROs in sputum samples with and without deduplication

In the sputum samples, the detection rates of CRKP, CRAB, CRPA, and MRSA without deduplication were significantly greater (28.39%, 76.19%, 46.95%, and 70.43%, respectively) than those with deduplication (19.99%, 63.00%, 38.05%, and 64.50%, respectively). The differences were statistically significant (all *P* < 0.05). The detection rate of CRECO without deduplication was slightly greater (3.67%) than that with deduplication (3.72%), but the difference was not statistically significant (*P* > 0.05). For more details, please refer to Table 3.

**Table 3:** Comparative analysis of the rates of MDRO detection in sputum samples with and without duplication

MDRO		Without deduplication	With deduplication	X^2^	P
CRKP	CRKP	592	283	31.797	< 0.001
	KP	2085	1416		
	Detection rate	28.39%	19.99%		
CRECO	CRECO	14	9	0.001	0.977
	ECO	381	242		
	Detection rate	3.67%	3.72%		
CRAB	CRAB	2048	858	77.652	< 0.001
	AB	2688	1362		
	Detection rate	76.19%	63.00%		
CRPA	CRPA	1133	508	27.669	< 0.001
	PA	2413	1335		
	Detection rate	46.95%	38.05%		
MRSA	MRSA	562	367	5.359	0.021
	SA	798	569		
	Detection rate	70.43%	64.50%		

*CRKP* Carbapenem-resistant Klebsiella pneumoniae, *KP* Klebsiella pneumoniae, *CRECO* Carbapenem-resistant Escherichia coli, *ECO* Escherichia coli, *CRAB* Carbapenem-resistant Acinetobacter baumannii, *AB* Acinetobacter baumannii, *CRPA* Carbapenem-resistant Pseudomonas aeruginosa, *PA* Pseudomonas aeruginosa, *MRSA* methicillin-resistant Staphylococcus aureus, *SA* Staphylococcus aureus

### Comparative analysis of the rates of MDRO detection in urine samples with and without duplication

There was a statistically significant difference in the CRKP detection rates in urine samples between the non-deduplicated (30.05%) and deduplicated (21.56%) groups (*P* < 0.05). For more details, please refer to Table 4.

**Table 4:** Comparative analysis of the rates of MDRO detection in urine samples with and without deduplication

MDRO		Without deduplication	With deduplication	X^2^	P
CRKP	CRKP	116	69	6.521	0.011
	KP	386	320		
	Detection rate	30.05%	21.56%		
CRECO	CRECO	23	18	0.248	0.618
	ECO	1258	1150		
	Detection rate	1.83%	1.57%		
CRAB	CRAB	26	24	0.053	0.818
	AB	75	73		
	Detection rate	34.67%	32.88%		
CRPA	CRPA	33	28	0.085	0.770
	PA	146	116		
	Detection rate	22.60%	24.14%		
MRSA	MRSA	20	18	0.067	0.800
	SA	53	51		
	Detection rate	37.74%	35.29%		

*CRKP* Carbapenem-resistant Klebsiella pneumoniae, *KP* Klebsiella pneumoniae, *CRECO* Carbapenem-resistant Escherichia coli, *ECO* Escherichia coli, *CRAB* Carbapenem-resistant Acinetobacter baumannii, *AB* Acinetobacter baumannii, *CRPA* Carbapenem-resistant Pseudomonas aeruginosa, *PA* Pseudomonas aeruginosa, *MRSA* methicillin-resistant Staphylococcus aureus, *SA* Staphylococcus aureus

### Comparative analysis of the detection rates of MDROs in blood samples with and without duplication

In blood samples, there was no significant difference in the detection rates of CRKP, CRECO, CRAB, CRPA, or MRSA between the samples without deduplication (46.77%, 3.37%, 92.86%, 29.41%, and 44.66%, respectively) and those with deduplication (46.88%, 4.08%, 89.74%, 36.67%, and 45.45%, respectively) (all *P* > 0.05). For more detailed information, please refer to Table 5.

**Table 5:** Comparative analysis of the rates of MDRO detection with and without deduplication in blood samples from patients

MDRO		Without deduplication	With deduplication	X^2^	P
CRKP	CRKP	123	60	0.000	0.984
	KP	263	128		
	Detection rate	46.77%	46.88%		
CRECO	CRECO	10	6	0.145	0.704
	ECO	297	147		
	Detection rate	3.37%	4.08%		
CRAB	CRAB	65	35	0.321	0.719
	AB	70	39		
	Detection rate	92.86%	89.74%		
CRPA	CRPA	15	11	0.456	0.499
	PA	51	30		
	Detection rate	29.41%	36.67%		
MRSA	MRSA	46	25	0.009	0.924
	SA	103	55		
	Detection rate	44.66%	45.45%		

*CRKP* Carbapenem-resistant Klebsiella pneumoniae, *KP* Klebsiella pneumoniae, *CRECO* Carbapenem-resistant Escherichia coli, *ECO* Escherichia coli, *CRAB* Carbapenem-resistant Acinetobacter baumannii, *AB* Acinetobacter baumannii, *CRPA* Carbapenem-resistant Pseudomonas aeruginosa, *PA* Pseudomonas aeruginosa, *MRSA* Methicillin-resistant Staphylococcus aureus, *SA* Staphylococcus aureus

### Comparative analysis of the rates of MDRO detection in BALF samples with and without duplication

The detection rates of CRKP and CRPA in BALF specimens were greater when deduplication was not performed (39.78% and 53.23%, respectively) than when it was performed (31.62% and 42.20%, respectively). These differences were statistically significant (all *P* < 0.05). For more details, please refer to Table 6.

**Table 6:** Comparative analysis of the rates of MDRO detection in BALF samples with and without duplication

MDRO		Without deduplication	With deduplication	X^2^	P
CRKP	CRKP	74	43	10.406	< 0.001
	KP	186	136		
	Detection rate	39.78%	31.62%		
CRECO	CRECO	0	0	/	> 0.999
	ECO	29	25		
	Detection rate	0	0		
CRAB	CRAB	353	213	1.219	0.270
	AB	396	247		
	Detection rate	89.14%	86.23%		
CRPA	CRPA	132	73	13.331	< 0.001
	PA	248	173		
	Detection rate	53.23%	42.20%		
MRSA	MRSA	38	33	0.11	0.740
	SA	54	49		
	Detection rate	70.37%	67.35%		

*CRKP* Carbapenem-resistant Klebsiella pneumoniae, *KP* Klebsiella pneumoniae, *CRECO* Carbapenem-resistant Escherichia coli, *ECO* Escherichia coli, *CRAB* Carbapenem-resistant Acinetobacter baumannii, *AB* Acinetobacter baumannii, *CRPA* Carbapenem-resistant Pseudomonas aeruginosa, *PA* Pseudomonas aeruginosa, *MRSA* Methicillin-resistant Staphylococcus aureus, *SA* Staphylococcus aureus, *BALF* Bronchoalveolar lavage fluid

## Discussion

In this study, we showed the requirements that are used to decide whether to deduplicate vary based on related standards, guidelines, and monitoring systems. In related studies, there was also a lack of consistency in the analysis of MDRO detection rates. Many studies [13, 23–26] have adhered to the practice of deduplication when determining MDRO detection rates, whereas other studies [27] have not employed deduplication. Moreover, the results of a previous study in which researchers did not perform deduplication were compared with data from another study [28] in which the results had undergone deduplication, and the phenomenon of comparing results subjected to deduplication with those not subjected to deduplication, or vice versa, is concerning. This lack of consistency is also a problem in practical infection control; some units or studies refer to the requirements of quality control indicators [11] to determine the detection rates (without deduplication) but compare the results with those of CHINET [12] (which requires deduplication). The lack of uniform monitoring standards raises questions about comparability. These questions warrant further consideration and study.

The results indicated that the inclusion of all specimens resulted in significantly greater rates of detection of CRKP, CRAB, CRPA, and MRSA in the absence of deduplication (33.52%, 77.24%, 44.56%, and 56.58%, respectively) compared to that with deduplication (24.78%, 66.25%, 36.24%, and 50.83%, respectively) (all *P* < 0.05). Specifically, in sputum samples, the detection rates of CRKP, CRAB, CRPA, and MRSA without deduplication (28.39%, 76.19%, 46.95%, and 70.43%, respectively) were significantly greater than those with deduplication (19.99%, 63.00%, 38.05%, and 64.50%, respectively) (all *P* < 0.05). In the urine samples, the detection rate of CRKP without deduplication (30.05%) was significantly greater than that with deduplication (21.56%) (*P* < 0.05). In BALF samples, the detection rates of CRKP and CRPA without deduplication (39.78% and 53.23%, respectively) were significantly greater than those with deduplication (31.62% and 42.20%, respectively) (all *P* < 0.05). This study revealed that when sputum, urine, and BALF samples are included, duplication removal has a significant impact on the MDRO rates of detection. Sputum samples are easily collected and are important for diagnosing lower respiratory tract infections [29, 30]. The use of a fibreoptic bronchoscope has made collecting BALF samples more convenient, and these samples are crucial for diagnosing and treating lower respiratory tract infections [31, 32]. Due to the extended treatment duration required for lower respiratory tract infections, multiple collections of sputum and BALF samples may be necessary for diagnosis, treatment, and evaluating treatment effectiveness [32, 33]. Similarly, urine culture specimens are easily obtained, and repeated sampling is often required to evaluate the effectiveness of treatment. Consequently, the same MDRO may be detected multiple times in lower respiratory tract samples and midstream urine specimens from the same patient. If duplicates are not removed during counting, the detection rates of most MDROs will be artificially inflated.

In this study, we also demonstrated a distinction in blood culture samples. The detection rates of CRKP, CRECO, CRAB, CRPA, and MRSA were 46.77%, 3.37%, 92.86%, 29.41%, and 44.66%, respectively, when deduplication was not applied. With respect to deduplication, the rates were 46.88%, 4.08%, 89.74%, 36.67%, and 45.45% for CRKP, CRECO, CRAB, CRPA, and MRSA, respectively. Thus, there was no significant difference between the results of these two methods (all *P* > 0.05). This finding is consistent with CHINET reports [12], which exclude duplicate strains isolated from the same patient (except in blood culture samples). Bloodstream infection is a common systemic disease in clinical practice, and a domestic meta-analysis revealed that its overall mortality rate is 28.7% [34]. Early and appropriate empirical antibiotic treatment is necessary [35]. Blood culture samples, as sterile fluids, are crucial for diagnosing bloodstream infections and are necessary for targeted antibiotic therapy [36]. The danger of bloodstream infections makes removing pathogenic bacteria as soon as possible a priority for medical staff, and the blood itself has a strong immune clearance ability, which can explain why there is no significant impact on whether MDROs are duplicated in blood samples.

### Limitations

Our research has two limitations. First, in this study, we did not conduct a stratified analysis based on factors such as age or sex, thus preventing us from determining specific population characteristics. Second, as this was a single-centre study, validation through multicentre studies is needed. Furthermore, in this study, we only clarified whether duplication affects the detection rates of MDROs according to the monitoring results. However, there are different methods of deduplication employed in practice. Determining whether different deduplication methods impact monitoring results requires further investigation. Regardless, the findings of this study suggest that infection prevention and control and bacterial resistance surveillance organizations should unify monitoring requirements when establishing relevant norms and standards to ensure that monitoring data are comparable and instructive.

## Conclusions

In conclusion, the impact of deduplication on the detection rate of MDROs varies depending on the type of specimen. Deduplication processing had a significant effect on the results for sputum, urine, and BALF samples. However, there was no statistically significant difference in the detection rates of blood samples with or without deduplication. When collecting, analysing, and comparing MDRO detection rates, relevant departments and medical institutions should ensure the consistency of monitoring standards to improve the quality of infection prevention and control monitoring. We call on the Infection Prevention and Control Organization to align its analysis guidelines with those of the Bacterial Resistance Surveillance Organization when monitoring MDRO detection rates.

## Data Availability

No datasets were generated or analysed during the current study.
